# Cohesin Is Dispensable for Centromere Cohesion in Human Cells

**DOI:** 10.1371/journal.pone.0000318

**Published:** 2007-03-28

**Authors:** Laura A. Díaz-Martínez, Juan F. Giménez-Abián, Duncan J. Clarke

**Affiliations:** 1 Department of Genetics, Cell Biology and Development, University of Minnesota Medical School, Minneapolis, Minnesota, United States of America; 2 Proliferación Celular, Centro de Investigaciones Biológicas, Consejo Superior de Investigaciones Científicas (CSIC), Madrid, Spain; Duke University Medical Centre, United States of America

## Abstract

**Background:**

Proper regulation of the cohesion at the centromeres of human chromosomes is essential for accurate genome transmission. Exactly how cohesion is maintained and is then dissolved in anaphase is not understood.

**Principal Findings:**

We have investigated the role of the cohesin complex at centromeres in human cells both by depleting cohesin subunits using RNA interference and also by expressing a non-cleavable version of the Rad21 cohesin protein. Rad21 depletion results in aberrant anaphase, during which the sister chromatids separate and segregate in an asynchronous fashion. However, centromere cohesion was maintained before anaphase in Rad21-depleted cells, and the primary constrictions at centromeres were indistinguishable from those in control cells. Expression of non-cleavable Rad21 (NC-Rad21), in which the sites normally cleaved by separase are mutated, resulted in delayed sister chromatid resolution in prophase and prometaphase, and a blockage of chromosome arm separation in anaphase, but did not impede centromere separation.

**Conclusions:**

These data indicate that cohesin complexes are dispensable for sister cohesion in early mitosis, yet play an important part in the fidelity of sister separation and segregation during anaphase. Cleavage at the separase-sensitive sites of Rad21 is important for arm separation, but not for centromere separation.

## Introduction

Faithful chromosome segregation requires that sister DNA molecules remain attached to one another until anaphase. This cohesion allows eukaryotic cells to unambiguously identify chromatids as sisters, attach paired sister chromatids to the mitotic spindle in a bioriented arrangement and to finally partition them among the daughter cells. Thus, without cohesion chromosome segregation would occur randomly in mitotic cells. Two mechanisms contribute to sister chromatid cohesion. Cohesin complexes composed of at least three protein subunits, Rad21/Mcd1/Scc1, Smc1 and Smc3, form a ring-like structure that is thought to encircle DNA [1]. In one model, two cohesin complexes, each trapping one sister DNA molecule, bind to each other to form a cohesive unit [2]. The association alone of cohesin with DNA is not sufficient for cohesion [3]–[5] and a number of other factors that are thought to license or establish cohesin-based cohesion have been described [6]–[14]. These include acetyl-transferases, cohesin binding proteins and, of particular interest, replication fork proteins [14]. Although the steps that accomplish cohesin licensing are yet to be described in detail, the evidence suggests that these mechanisms are biochemically linked to DNA replication.

It is the double-helical nature of DNA itself that, when replicated semi-conservatively, produces the second mechanism of sister chromatid cohesion: DNA catenation. Exactly because DNA strands are coiled around each other, their replicative products, the sister DNA molecules, also become physically intertwined [15]. Unlike cohesin, whose licensing is coupled to DNA duplication by biochemical means, DNA catenation is an inevitable outcome of DNA replication because these processes are physically coupled. The strand passage reaction performed by type II DNA topoisomerases [16], [17] resolves the numerous sister chromatid catenations that arise during S-phase, many being removed before mitosis [18] but some remaining at centromeres and along chromosome arms until the onset of anaphase [19]–[21]. How removal of the catenations is selectively controlled is not understood [22]–[24].

Similar to the step-wise removal of DNA catenations, at least two pools of cohesin are lost from chromosomes as cells traverse mitosis [25]–[27]. Most chromosomal cohesin dissociates in prophase [5], [27]–[29], but some cohesin between chromosome arms and at the centromere regions is thought to be protected from removal by the so-called prophase pathway. One factor that appears to be essential for this protection of some of the chromosomal cohesin is hSgo1, a protein found at centromeres in mitosis [30]–[37]. Anaphase initiation in human cells correlates with separase-mediated cleavage of a small pool of Rad21, that would presumably open the cohesin ring, releasing it from the DNA. Furthermore, this cleavage of Rad21 coincides with a sudden lack of detectable Rad21 seen at human centromeres in metaphase, suggesting that these Rad21 molecules, that probably dissociate from centromeres, might be the same molecules cleaved by separase [27].

These studies provided circumstantial evidence linking Rad21 cleavage at centromeres to centromere separation, but provided no direct test of whether this cleavage is a prerequisite for anaphase initiation or centromere separation. Other studies have, however, described more compelling evidence in favor of this hypothesis. Expression of a non-cleavable Rad21 mutant in human cells was observed to cause a failure in anaphase chromatid disjunction, though it was not determined whether this failure to disjoin chromatids was due to remaining arm or centromere cohesion [38]. Other studies have examined MEFs from separase knock-out mice, finding that chromatid disjunction was blocked without separase, but again it was not determined whether chromosome arms or the centromeres were unable to separate [39], [40]. Depletion of separase by RNAi resulted in similar cut-telophase phenotypes, but in many anaphase-like cells it was the chromosome arms, not the centromere regions, that failed to separate [41]. Based on these studies it remained open to question what are the roles of separase and cohesin in the regulation of centromere separation in human cells.

The above studies did indicate, however, that cohesin cleavage might be essential for anaphase chromatid disjunction. Conversely, recent work described the role of cohesin in sister chromatid cohesion [7], [11], [42]–[44]. These studies, in human cells and *Xenopus* egg extracts, used RNA interference (RNAi) or antibodies to selectively deplete Rad21. While some of the observations made were in agreement, an important conclusion stemming from the studies in egg extracts was incongruent with the human cell studies which claimed that cohesin is essential for sister chromatid cohesion. In *Xenopus* egg extracts, cohesin depletion did not disrupt centromere cohesion unless chromosomes were bioriented on the mitotic spindle, indicating that another cohesive force is capable of preserving centromere cohesion as long as spindle tension is absent [43].

Here we report that cohesin is dispensable for sister centromere association in human cells. Similar to the findings in *Xenopus* egg extracts, we did not observe loss of centromeric cohesion in prophase or prometaphase cells. Most cells were in fact able to form normal metaphase plates, indicating functional cohesion at the centromeres. Anaphase occurred aberrantly, however, reflecting a similar requirement for cohesin, as seen in egg extracts, for coordinated separation of the sister centromeres upon spindle attachment and congression to the metaphase plate. In cells expressing non-cleavable Rad21, we also observed aberrant anaphases, but it was the separation of chromosome arms that was perturbed, rather than separation of sister centromeres. We conclude that separation of chromosome arms is promoted by Rad21 cleavage and that cohesin-independent cohesive forces maintain cohesion at centromeres until anaphase.

## Results

### Cells with cohesion defects do not accumulate in mitosis in the absence of Rad21 or Smc3

**Figure 1:**
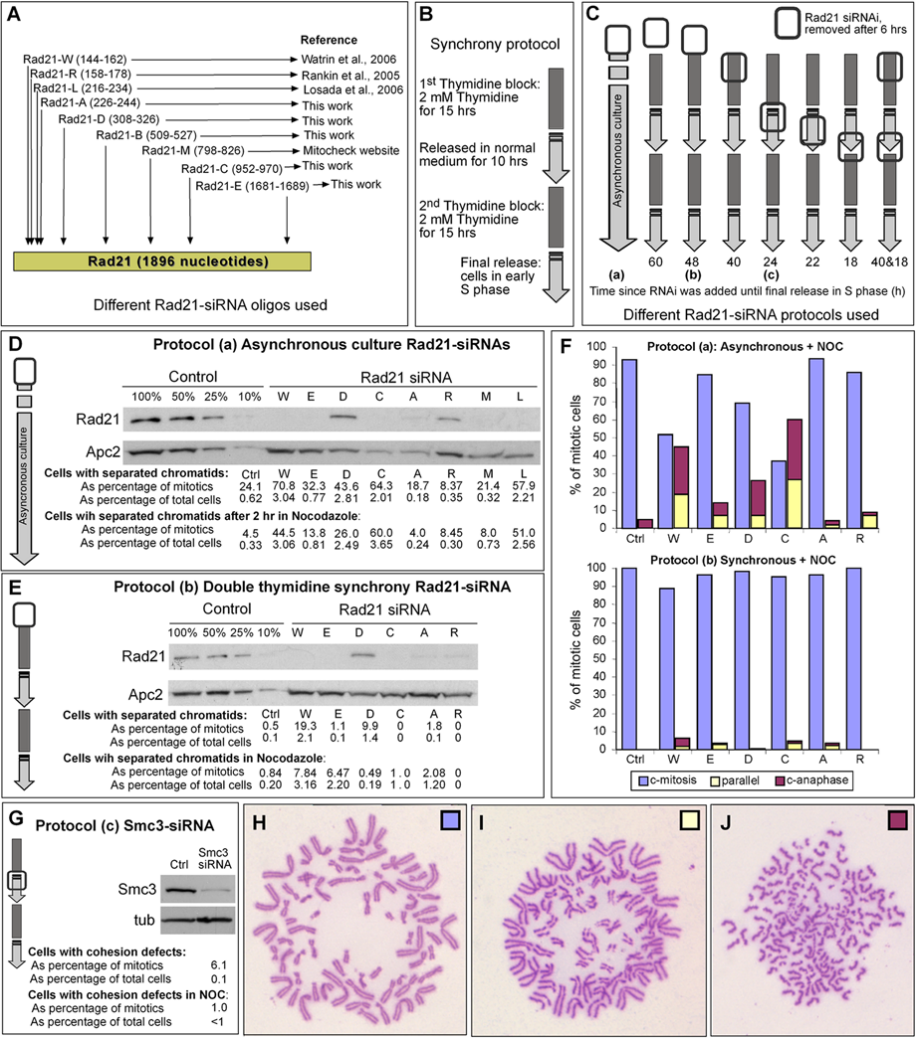
Rad21 depletion in HeLa cells. (A) Cartoon showing locations in the Rad21 mRNA sequence that were targeted by 9 different siRNA oligos used in this study. (B) Protocol used for achieving early S-phase synchrony of HeLa cells. (C) Summary of 8 different protocols used to deplete Rad21. Open rectangle indicates the timing of siRNA transfection relative to the synchrony protocol (which was done as described in B) or in asynchronous culture (leftmost arrow). Protocol-A, -B and -C are indicated as (a, b, c). For the protocols involving synchrony, the numbers at the bottom of each protocol indicate the total time from the siRNA transfection until the cells were released from the final early S-phase arrest, at which time protein extracts were prepared for biochemical analysis. In parallel, samples were fixed with Carnoy's for cytological analysis 11 hours after the release (in half of the samples, nocodazole was present during the last 2 hours before Carnoy's fixation). For the asynchronous protocol, protein samples and Carnoy's fixed samples were prepared 48 hours after siRNA transfection (in half of the samples, nocodazole was present for 2 additional hours and thus the cells were fixed with Carnoy's 50 hours after siRNA transfection). (D,E) Western blots showing degrees of Rad21 depletion achieved using Protocol-A and Protocol-B. Apc2 is a loading control. Left four lanes are dilution series of control samples. Letters above the other lanes denote the siRNA oligos used and correspond to those depicted in A. (F, H–J) Cytological analysis corresponding to the biochemical analysis shown in D and E. Samples were scored to determine the frequencies of mitotic cells that had cohered sisters (H), resolved primary constrictions (I) or fully separated sister chromatids (J). (Note that the cells shown are not arrested in nocodazole, and therefore serve as examples only. Examples with nocodazole are shown in Fig. 2.) The data tabulated in D and E indicate the frequencies of sister separation (combining the categories depicted in I and J) and the histogram plots (F) are colored corresponding to the categories depicted in H–J. (G) Depletion of Smc3 in HeLa cells (Protocol-C). Western blot shows Smc3 level at the time of release from S-phase synchrony. Tub = alpha-tubulin loading control. Cells with separated sisters chromatids (in tabulated form) were quantified from chromosome preparations fixed with Carnoy's and stained with Giemsa.

In order to provide a comprehensive description of phenotypes resulting from cohesin depletion in HeLa cells, we performed experiments that used nine different siRNAs targeted at different regions of Rad21 (Fig. 1A) as well as siRNAs specific for Smc3 (see Materials and Methods). We tested eight different protocols in which either asynchronous cultures were transfected with siRNA, or cells were synchronized in combination with the siRNA transfection (Fig. 1B,C). Three of the siRNAs, herein referred to as Rad21-W, -L and -R, had been described in previous studies, and the asynchronous protocol that we employed (Protocol-A) replicated those studies with only minor modifications [7], [11], [44]. In Protocol-A, briefly, logarithmically growing HeLa cells were transfected with siRNA specific for Rad21 and 48 hours later, cells were either harvested immediately (time zero) or after a further 2 hours with nocodazole. After fixing with Carnoy's, the preparations were dropped onto microscope slides and stained with Giemsa. Judged by Western blotting, 6/8 of the siRNAs efficiently depleted Rad21 to less than 10% of the endogenous level (Fig. 1D), similar to the published studies where Rad21 was depleted by at least 70–90% [7], [11], [44]. Also in agreement with the published studies, we observed that 30–60% of the mitotic cells possessed chromosomes with partly or fully separated sister chromatids, following the 2 hour nocodazole treatment (Fig. 1F, top panel). This phenotype was categorized into cells with completely separated sister chromatids (“separated”), or cells with sisters in which the primary centromeric constriction was absent, but the sisters remained positioned in close proximity (“parallel”) (Fig. 1F,H–J).

**Figure 2:**
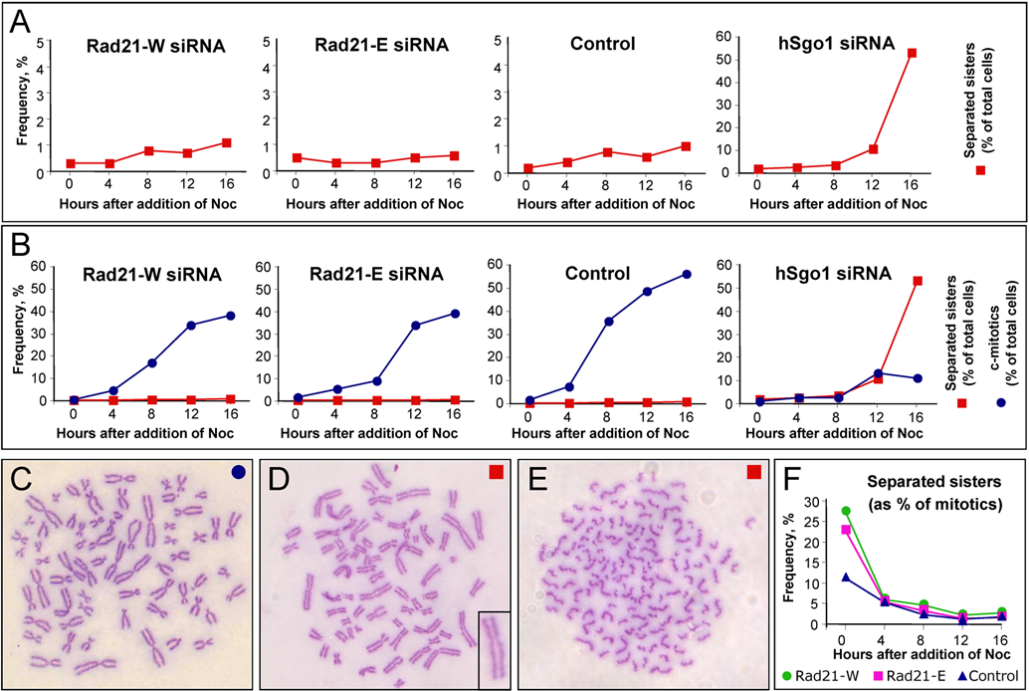
Rad21 depleted cells do not separate their sister chromatids in the presence of nocodazole. (A–E) HeLa cells synchronized in early S-phase and depleted of Rad21 (or mock depleted) with the indicated siRNA oligos as described in Figure 1, Protocol-B. Nocodazole was added after release from the S-phase synchrony and cells prepared for cytological analysis at the indicated time points. Cells with separated or partly separated sister chromatids were scored versus cells with cohered sisters, as depicted in C–E. For comparison, Sgo1-depleted cells were examined under the same conditions. Sgo1 depletion (∼80% depletion) was slightly less efficient that Rad21 depletion (data not shown). Note that the data in panel A are reproduced in panel B but with a different Y-axis scale. (F) HeLa cells were depleted of Rad21 (or mock depleted) with the indicated siRNA oligos as described in Figure 1, Protocol-A. Nocodazole was added 48 hours after siRNA transfection and cells prepared for cytological analysis at the indicated time points, then scored as in A–E (% separated sisters combines the categories shown in D+E).

Superficially, our data are congruent with the published studies, but one observation prompted us to investigate this further. We noticed that there were higher frequencies of cells with separated sisters in the zero time point samples than in the samples that had been incubated with nocodazole for a further two hours (Fig. 1D). This indicated that the cells which accumulated in mitosis during the 2 hour nocodazole treatment must have reached mitosis with normal cohesion, thus reducing the overall % of mitotic cells that had separated sisters. If true, then it could have been the case that only a very minor fraction of the total population of cells displayed the sister separation phenotype. This was important to establish for two reasons. Firstly, when expressed in terms of a percentage of the whole population, rather than as a fraction of the mitotic cells, the frequency of cells with separated sister chromatids after Rad21 depletion was never greater than 4% with any of the siRNAs used (Fig. 1D). Second, previous Rad21 depletion studies had reported that in the cells with separated sister chromatids, a mitotic checkpoint became activated, preventing those cells from exiting mitosis [44]. Checkpoint activation would be an expected outcome of a cohesion defect since separated sisters would not be able to become bioriented on the spindle and therefore there would be no tension on the sister kinetochores. In this scenario, if Rad21 depletion leads to a cohesion defect, then cells with separated sister chromatids ought to accumulate over time whether or not nocodazole was present.

To test this hypothesis, we began by employing synchrony strategies that allowed Rad21 depletion combined with cell cycle arrest (Fig. 1B). Using this approach, we could accumulate most of the cells in mitosis over a short time-course after nocodazole addition. It could have been the case that the timing of Rad21 depletion, relative to cell cycle synchronization, produced different phenotypes; therefore, we tested seven different protocols, varying the timing of synchrony versus siRNA transfection (Fig. 1C). Depletion of >90% of Rad21 was observed by 18 hours after transfection (following the shortest protocol). Compared with the shortest protocol employed, in the longest of the protocols Rad21 siRNA was transfected more than a full cell cycle sooner (42 hours) to ensure absence of Rad21 at the time that DNA replication was initiated in S phase. Furthermore, in one protocol the HeLa cells were transfected twice in an attempt to increase the efficiency of the knock-down. In most cases, Rad21 depletion was at least as efficient as described above using Protocol-A with asynchronous cultures (Fig. 1E and data not shown). After release from the early S-phase synchronies, nocodazole was added and the cells collected at the next mitosis after 16 hours (In control and Rad21-depleted cells, the peak of the mitotic wave was between 10 and 12 hours after release). Since all of the synchrony protocols resulted in very similar phenotypes, regardless of the timing of siRNA transfection, we present representative data corresponding to Protocol-B. Despite the efficient depletion of Rad21 produced by most of the combinations of synchrony protocol and the particular siRNA transfected, very low frequencies, a maximum of ∼8% of the nocodazole-arrested mitotic cells, with separated or parallel sister chromatids were observed (Fig. 1E,F). Expressed as a percentage of total cells, only about 2–3% of the Rad21-depleted cells separated their sister chromatids in the presence of nocodazole, compared to ∼1% in control-treated cells. Similarly, and in agreement with the experiments that used the asynchronous protocol (Protocol-A), very low frequencies of cells with separated sisters were observed in the absence of nocodazole (∼2% of total cells) (Fig. 1E). These findings held true in experiments where Smc3 was depleted (Fig. 1G). Therefore, after depletion of cohesin proteins by >90% by the time of S phase progression, most cells reached metaphase with normally cohered sister centromeres and even upon arrest induced by nocodazole cohesion was maintained. Perhaps even more telling was the fact that there was no increase in the frequency of mitotic cells with separated sister chromatids when we employed protocols that extended the time interval between siRNA transfection and nocodazole addition.

### Cells with cohesion defects are not eliminated by apoptosis or mitotic slippage after Rad21 depletion

The surprising results described above suggested that cohesin depletion only leads to arrest in mitosis with separated sister chromatids in a small fraction of the cell population. Another explanation could be that mitotic cells with separated sisters were selectively depleted during the time-courses, for example by mitotic apoptosis or by mitotic slippage [45], where cells return to interphase. We therefore repeated these time-course experiments, but taking samples every 4 hours after release from early S-phase synchrony. Representative examples are shown in Figure 2 that used Rad21-W and Rad21-E siRNAs. In agreement with the above data, mitotic cells with separated sisters were infrequently observed at any time-point, not reaching more than 2% of the total cells in this series of experiments (Fig. 2). Although the cells treated with Rad21-specific siRNA typically entered mitosis with slightly delayed kinetics relative to the control-treated cells, the frequency of apoptotic cells was not different than controls (data not shown). The frequency of cells with restitution nuclei (indicative of mitotic slippage) was also similar to controls and never exceeded 0.2%. For further comparison, we depleted hSgo1 from HeLa cells, as previously reported [35], and under the same experimental conditions of cell cycle synchrony we observed that more than 50% of the total cells (or 83% of the mitotic cells) had fully separated sister chromatids at the 16 hour time point. Thus, although hSgo1-depleted cells had a dramatic defect in the ability to hold sisters together, Rad21-depleted cells did not.

All of the above data are consistent with depletion of Rad21 resulting in the arrest of only a minor fraction of cells in mitosis with separated sister chromatids. If this were equally true when asynchronous or synchronous cultures were treated with different Rad21-specific siRNAs, regardless of the experimental protocol, then we ought to observe a decrease in the frequency of mitotic cells with separated sister chromatids, over time, when cells subjected to Protocol-A (asynchronous) were incubated with nocodazole. As shown in Figure 2F, this indeed was the case when using either Rad21-W or Rad21-E. This experiment also ruled out the possibility that Rad21-depleted cells might have tended to separate their sisters more quickly than control-treated cells upon nocodazole arrest. We conclude that consistent results are obtained regardless of cell cycle synchrony and with most of the siRNAs tested. Moreover, our data are not inconsistent with the published data: we observed similar frequencies of mitotic cells with separated sister chromatids using Protocol-A, the protocol used in previous studies [7], [11], [44], and if cells were harvested after short periods of incubation with nocodazole. However, further nocodazole incubation revealed that all of the newly arrived mitotic cells must have had cohered sister chromatids, because the frequency of cells with separated sisters dropped strikingly over time.

### Cells with separated sister chromatids after Rad21-depletion most likely arise after aberrant anaphase attempts

The low incidences of mitotic arrest with separated sister chromatids could be explained in two ways. Depletion of Rad21 even exceeding 90% may have been rarely sufficient to allow sister separation, since only a small number of centromeric cohesin complexes might be needed for centromeric cohesion. On the other hand, it is a challenge to argue that, under these conditions, almost all cells could possess chromosomes with cytologically normal primary constrictions [46], given that the centromere regions of human chromosomes contain megabases of DNA. Some cytological defect at the centromere ought to have been observed in the Rad21-depleted cells. Furthermore, some differences would have been expected depending on the timing of Rad21 siRNA transfection, since earlier transfection of the siRNA would increase the proportion of cells lacking Rad21, or the efficiency of depletion, before the cells were released from the S-phase synchrony. An alternative and simple explanation is that cohesin complexes act redundantly with another cohesive force, namely DNA catenations. Sister DNA duplexes become physically interlocked during their replication as a consequence of the double-helical nature of DNA. Many DNA catenations are resolved before and during the early stages of mitosis, but just before anaphase onset, some catenations remain at centromere regions. This is evident as inhibition of DNA topoisomerase II, the eukaryotic enzyme that decatenates sister DNA molecules, in late metaphase causes a complete block of centromere separation [19], [21]. In mitosis, topoisomerase II appears to be targeted to centromere regions by a process that requires sumoylation by PIASγ [23]. In cells depleted of PIASγ, metaphase delays are observed and sister chromatids fail to separate even when cohesin complexes are removed from the chromosomes by co-depletion of hSgo1. In these chromosomes, Rad21 cannot be detected (by immuno-staining), but the primary constriction appears cytologically normal and catenations remain [23]. These data provide some evidence that the primary constriction, and therefore centromeric cohesion, is maintained by DNA catenations in the absence of cohesin. Applying this line of reasoning to the present study, catenations might provide centromere cohesion after Rad21 depletion. For this reason, it became important to determine the origin of those cells depleted of Rad21 that did arrest in mitosis with separated sister chromatids.

**Figure 3:**
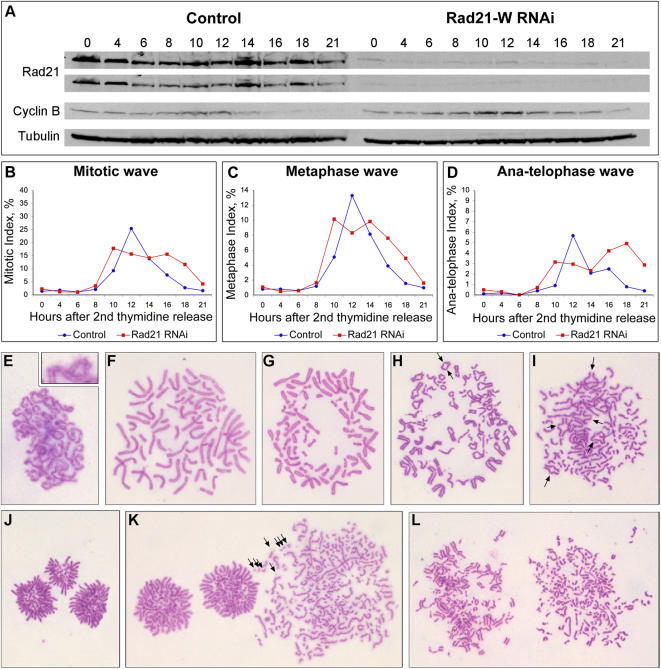
Time course of synchronous Rad21-depleted HeLa cells (Rad21-W siRNA). HeLa cells were synchronized by double thymidine block and transfected (or mock transfected) with Rad21-W siRNA according to Protocol-C (see Figure 1). After release from the early S-phase synchrony, samples were taken for biochemical (A) and cytological (B–L) analysis over the next 21 hours. (A) Western blot showing level of Rad21 depletion and mitotic status of the cells based on cyclin B levels (Tubulin = loading control). (B–D) Samples for cytology were fixed and stained (see Material and Methods) and mitotic categories scored on at least 1000 cells per sample and time point. (E–F) Cytological features of cells transfected with Rad21-W siRNA: (E) Normal sister cohesion upon nuclear envelope breakdown (observed in 100% of the prophase and prometaphase cells); (F) Normal sister cohesion in early prometaphase; (G) Normal metaphase plate formation; (H–I) Aberrant anaphases – centromere regions separating before arms (arrows) and some chromosomes segregating to the poles before other chromosomes have separated their sisters. (J) Abnormal telophase – chromosomes have segregated (unevenly) to three cell poles; (K) Chromosome breaks in a cell with separated sister chromatids (presumably a post-anaphase cell); (L) Chromosome breaks in metaphase or early anaphase cells.

**Figure 4:**
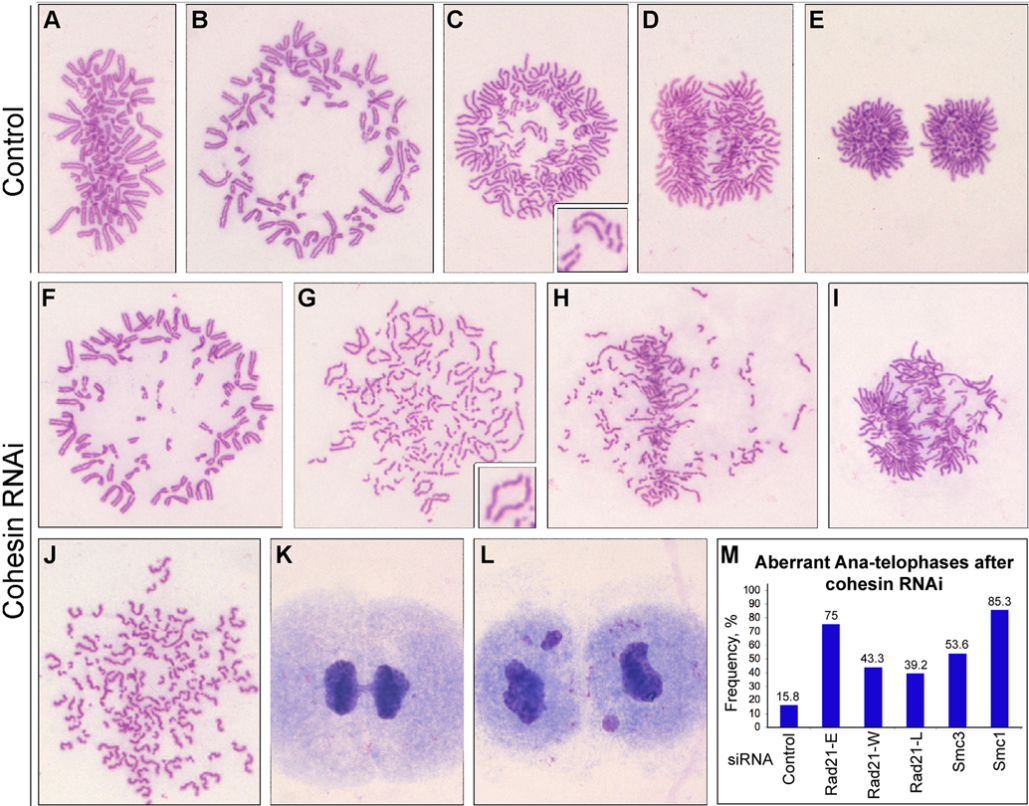
Anaphase and telophase abnormalities in Rad21-depleted HeLa cells. (A–L) Cells fixed with Carnoy's and stained with Giemsa. (A–E) Control series showing the cytology of control-treated cells at different mitotic stages: (A,B) Metaphases - side and polar views; (C) Early anaphases – polar and side views; (E) Telophase. (F–L) Examples of cells treated with Rad21-specific siRNA: (F) Metaphase – polar view; (G–I) Aberrant anaphase – asynchronous anaphase, defined as described in Fig. 3; (J) Apolar telophase – separated sisters scattered in the cytoplasm (cell may be arrested in mitosis based on the level of chromosome condensation); (K–L) Telophases with chromosome bridges and lagging chromosomes; (M) Quantification of abnormal anaphase and telophase cells in control-treated and cohesin-depleted cells. At least 1000 cells were scored per sample.

To this end, and to gain a more complete picture of the phenotype of cells depleted of Rad21, we performed detailed time course experiments after early S-phase synchrony, using Rad21-E, Rad21-W and Rad21-L siRNAs or siRNA specific for Smc3, taking samples every two hours for biochemical and cytological analysis. The major characteristic phenotypes described below were observed in all four cases (Fig. 3 and Fig. S1, S2, S3). After release from early S-phase, the wave of mitotic cells appeared with slightly delayed kinetics after Rad21 depletion and was typically somewhat broader than in control-treated cells (Fig. 3B). This was evident also in Western blots, which typically revealed a slight delay in the appearance and subsequent decay of mitotic proteins such as cyclin B1 and phospho-H3 (Fig. 3A, S1A, S2A, S3A). Consistent with the studies described above, prophase and early prometaphase Rad21-depleted cells always had cohered sister chromatids (Fig. 3E), as did mid-late prometaphase (Fig. 3F) and metaphase cells (Fig. 3G). In these cells, the primary centromeric constriction was always well defined, as in control-treated cells. That a wave of cells passing through metaphase (Fig. 3C) could be clearly observed in these time-courses indicates that most cells reached metaphase with normally cohered sisters. However, the metaphase and anaphase/telophase waves were broader than in controls, indicating defects in progression though these mitotic stages (Fig. 3C,D). We did not observe any obvious differences between the control-treated and Rad21-depleted metaphase cells, but abnormal anaphases and telophases were observed at an increased frequency (Fig. 3H–J). Further examples of these phenotypes are compared with control-treated cells in Figure 4 and quantified in Figure 4M. In the control-treated HeLa cells, 84% of the anaphases were cytologically normal (Fig. 4C–E,M). Aberrant anaphases fell into two major categories in the Rad21-depleted cells, those in which sister separation occurred asynchronously, and those that possessed extra spindle poles (this latter category is discussed elsewhere). In addition, mitotic cells were seen with broken chromosomes after depletion of Rad21 (Fig. 3K,L and Fig. S4).

Asynchronous anaphases were characterized by two phenomena: sister centromeres sometimes separated before chromosome arms (Fig. 3H, 4G) and the sisters of some chromosomes separated fully before those of other chromosomes (Fig. 3H,I, 4H). Such cells often had identifiable metaphase plates with some chromosomes, that usually were in the process of separation, positioned close to the plate, as well as some fully separated sisters that were closer to the spindle poles (Fig. 4H and Fig. S1I are good examples). We posit that these phenotypes are the consequence of an asynchronous attempt at anaphase chromosome segregation. Published studies had described very similar human cells depleted of Rad21, from a cytological point of view, but interpreted such cells as being prometaphases with precociously separated sister chromatids. Taking all of the available data together, we suggest that these are pseudo-anaphase cells, based on the following information: (1) They appeared to be cyclin B and securin positive, indicating that the APC/C was inactive [7], [44], but (2) the chromosomes with separated centromeres lacked MCAK, CENP-E and Aurora B at their kinetochores [11], [44] indicating checkpoint inactivation, and (3) such cells had elongated mitotic spindles [7], [11] indicating an attempt at anaphase. Because we observed a metaphase wave in our synchronous time-course experiments, no more prometaphases than in control cells, and no cells in the process of breaking down the nuclear envelope with defects in cohesion between sisters (Fig. 3E, S1E, S2E, S3E), it is unlikely that the cells with separated sister chromatids were prometaphases with precociously separated sisters. However, we note that our cytological method of Carnoy's fixation and chromosome spreading is not optimal for distinguishing metaphase cells from very late prometaphase cells that possess a small number of non-congressed chromosomes. Regardless of this, our analysis has revealed that in the vast majority of cells, most, if not all, chromosomes reach the metaphase plate with cohered centromeres. At very low frequencies, cells were observed in which all of the sister chromatids were fully separated, but were not arranged around separated poles (Fig. 4J) as is typically seen in telophase cells (Fig. 4E). These cells could have been arrested in mitosis, judging by the degree of chromosome condensation typically seen, but as this category did not accumulate during the time-course, and never exceeded 1–2% of the total cells, we suggest that such cells correspond to those cells with separated sister chromatids seen in the experiments using asynchronous cultures (Fig. 1). Such cells could have separated their sisters upon nuclear envelope breakdown, or could have become arrested in mitosis following anaphase attempts. To examine this further, on a cell by cell basis, we performed video time-lapse microscopy on HeLa cells depleted of Rad21 and expressing H2B-GFP. From 30 cells filmed that entered mitosis, only one cell became arrested in mitosis (see Fig. S5 and Movie S1). In this cell, an apparently normal metaphase plate formed but then anaphase onset did not appear to occur with the appropriate timing. Metaphase seemed to last 1–1.5 hours, after which chromosomes began slowly to leave the plate - possibly indicating an attempt at anaphase. From this point onwards, until the end of the movie (more than 15 hours later), the cell remained in mitosis. The frequency of cells that became arrested in mitosis in these time-lapse studies was therefore similar to the frequency of cells with fully separated sisters seen in the Giemsa-stained material. That this cell formed an apparently normal metaphase plate, argues that this category of the Rad21-depleted cells had cohered centromeres at least until metaphase (as otherwise chromosome biorientation could not take place), prior to an asynchronous attempt at anaphase and mitotic arrest.

To summarize these experiments, we find that most cells depleted of Rad21 reach metaphase, some appear to undergo an asynchronous anaphase, then exit mitosis. During cytokinesis, cells were sometimes observed with chromatin bridges or micronuclei (Fig. 4 K,L). At a low frequency, Rad21-depleted cells became arrested in mitosis and did not attempt cytokinesis. However, we found very few cells that arrested in mitosis with fully separated sister chromatids and cells with this phenotype did not accumulate over time after the siRNA treatment, either in the presence or absence of nocodazole. This was not because such cells exited mitosis, because the frequency of restitution nuclei observed in any of our Rad21-delpetion studies never exceeded 0.2%. The small number of cells observed that had fully separated sister chromatids may have arisen as a result of aberrant anaphase progression resulting in arrested apolar telophase cells. They are unlikely to have arisen due to precocious sister separation, before anaphase, because we did not observe any prophase or early prometaphase cells with separated sister centromeres after Rad21 depletion, and in live cell analysis by time lapse microscopy, mitotic arrest with separated sisters was preceded by metaphase plate formation indicating functional sister centromere cohesion.

### Non-cleavable Rad21 prevents chromosome arm but not centromere separation

**Figure 5:**
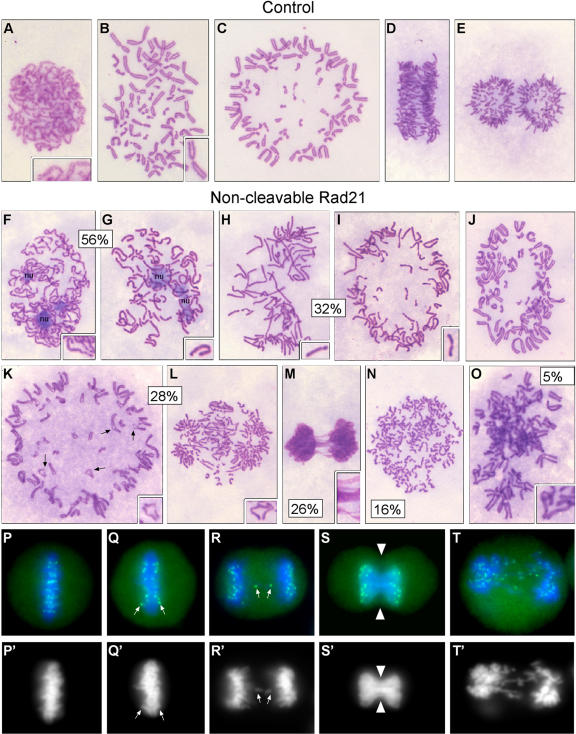
Cytological analysis of HeLa cells expressing non-cleavable (NC) Rad21. Control HeLa cells in which wild type MYC-Rad21 expression had been induced (A–E) were compared with HeLa in which NC-Rad21 (NC-MYC-Rad21) expression had been induced 72 hours prior to fixation and staining with Giemsa (F–O) or immuno-staining with CREST serum (P–T). Controls: (A,B) Normal cohesion and sister resolution in late prophase and early prometaphase; (C) Metaphase – polar view; (D) Anaphase -side view; (E) Telophase. NC-Rad21: (F–G) Delayed nucleolus disassembly in early prometaphase and delayed sister chromatid resolution (56% of prometaphases had the resolution defect); (H–I) Late prometaphase and metaphase cells with unresolved sisters (32%); (J) Metaphase – polar view; (K–L) Aberrant anaphases – centromere regions separating before arms (arrows) and some chromosomes segregating to the poles before other chromosomes have separated their sisters (28% were abnormal based on these criteria); (M) Cut telophase (26% of the telophases had this phenotype); (N) Apolar telophase (16% of the telophases had this phenotype); (O) Partially resolved diplochromosomes (5% of mitotics), in agreement with the described separase RNAi phenotype [41]; (P–T) DNA (DAPI; blue) and CREST (green) staining of kinetochores after NC-Rad21 induction. (P′–T′) DAPI channel only. (P) Metaphase; (Q) Early anaphase – one pair of kinetochores appears to be segregating prematurely; (R) Laggard kinetochores in anaphase; (S) Cut telophase - kinetochores have segregated to the poles despite deficient karyokinesis; (T) Bridged and laggard chromosomes in telophase.

These findings thus raised the possibility that cohesin is dispensable for centromere cohesion, presumably since DNA catenations are alone capable of providing a cohesive mechanism. The role of cohesin at centromere regions in human cells is ill-defined. Rad21 localizes to centromeres and between chromosome arms in metaphase cells and disappears at the time of anaphase onset [27]. However, there is no direct evidence that links cohesin with centromere association. Expression of non-cleavable Rad21 (resistant to separase) in human cells resulted in failed chromatid disjunction [38], but whether centromere separation, chromosome arm separation, or both, were prevented was not studied. Given the unexpected outcome of Rad21 depletion in human cells, we therefore examined the phenotypes resulting from expression of non-cleavable Rad21 (NC-Rad21) in more detail than had been done previously. For these studies, we used the cell line constructed by Hauf et al. (2001) and we reproduced the experimental conditions used previously so that our data could be directly compared with the published data. After induction of NC-Rad21, we harvested cells, fixed with Carnoy's or paraformaldehyde, and either stained with Giemsa or with CREST antiserum to visualize kinetochores (Fig. 5). As previously reported, NC-Rad21 resulted in failed chromosome segregation (cut-telophases) in ∼26% of the cells, but we also noticed additional phenotypes that are described here in detail. Two defects were observed in prophase and early prometaphase cells. Unlike in controls, where the nucleolus always disassembled in late prophase (Fig. 5A), NC-Rad21 late prophases and early prometaphases were seen with intact nucleoli (Fig. 5F,G). In addition, 56% of NC-Rad21 early prometaphases and 32% of late prometaphase-metaphase cells had unresolved sister chromatids (Fig. 5F–I). Since sister chromatid resolution normally takes place in late prophase (Fig. 5A) and is complete by early prometaphase (Fig. 5B) in mammalian cells [47], [48], NC-Rad21 appears to delay resolution of the chromosome arms. Consistent with these data, each of these phenotypes was previously seen in cells depleted of separase [41]. Most metaphase NC-Rad21 cells appeared normal at this level of resolution (Fig. 5J), but unexpectedly, 28% of anaphase cells had phenotypes consistent with centromere separation in the absence of complete chromosome arm separation (Fig. 5K,L). In these cells, centromeres were often clearly separated while the chromosome arms remained cohered (Fig. 5K). Also within this category of anaphase cell, it was clear that not all chromosomes separated their sister chromatids synchronously (Fig. 5L). The frequency of anaphase cells with these defects (28%) was similar to the frequency of cut-telophase cells with chromatin bridges (26%), suggesting that these uncoordinated attempts at anaphase can entirely account for the observed failures in sister chromatid disjunction. Visualization of kinetochores with CREST antiserum revealed similar findings in that cut-telophase NC-Rad21 cells were apparently able to separate their sister kinetochores, consistent with the notion that failed sister disjunction resulted from the inability to separate chromosome arms rather than centromere regions (Fig. 5P–T) [49]. From these studies we conclude that cleavage of cohesin Rad21 is important for the ability of chromosome arms to become resolved in preparation for anaphase and for timely separation of chromosome arms during anaphase. However, we find no evidence for a requirement of Rad21 cleavage for centromere separation in anaphase cells.

## Discussion

### Evidence that Rad21 cleavage is dispensable for centromere separation

Previous studies have provided evidence that the APC/C is not required for release of centromere cohesion in mammals. In HeLa cells, depletion of Apc2, an essential component of the catalytic center of the APC/C, results in highly aberrant anaphase progression, but does not prevent sister chromatid separation [35]. Strikingly similar phenotypes are observed in vivo in mouse hepatocytes, and in tissue culture in MEFs, after genetic deletion of APC2 induced by Cre-recombinase [35]. In each case, though these cells initiate anaphase, defined cytologically by sister chromatid separation [46], cyclin B remains stable and cells arrest in a pseudo-telophase state with fully separated sister chromatids. These data called into question whether APC/C activity, as well as cyclin B and securin degradation, are essential for sister separation.

Using more direct approaches to address these important issues, we and others provided evidence that neither non-degradable securin nor stable cyclin B could prevent sister centromere separation during mitosis [35], [50], [51]. Considering these experiments together, it remains possible that alternative mechanisms regulate separase, enabling its activation in the presence of securin or cyclin B. To clarify this point, we chose to directly examine the role of Rad21, an essential component of the cohesin complex in yeast and vertebrates, in maintaining centromere cohesion by expressing in HeLa cells a mutant form of Rad21 that cannot be cleaved by separase [38]. There are three sites in Rad21 that can be cleaved by separase; the mutant used here had mutations in all three sites [38]. As reported previously [38], induction of the non-cleavable Rad21 mutant interfered with mitosis (Fig. 5), perturbing cells in anaphase, presumably due to a lack of proper sister chromatid separation. However, when we examined the cause of the anaphase failures we observed that in almost all cells, centromere separation was not affected. Arm separation was strongly impaired in about 28% of the cells, producing a rhomboid chromosome appearance and accounting for the abortive anaphases (∼26%) that have been described before [38]. When expressed at a similar level, exogenous wild type Rad21 did not obviously affect arm resolution (Ref. 38 and data not shown). A minimal conclusion is that cleavage of Rad21 at the separase-sensitive sites is not needed for dissolution of centromere cohesion. Quite possibly, the integrity of the cohesin complex at centromere regions is compromised by another mechanism at the onset of anaphase. These data agree with and can explain the facts that the APC/C is dispensable for centromere separation [35] and that centromeres can separate in the presence of non-degradable securin and stable B-type cyclins [35], [50], [51]. An alternative possibility is that cohesion at centromere regions is not mediated by cohesin in mammals.

### Rad21 depleted cells maintain centromere cohesion in the presence of nocodazole

That Rad21 cleavage was apparently not required for centromere separation in human cells was in agreement with our observation that cohesin was dispensable for centromere cohesion before anaphase. We tested nine different siRNAs targeting different regions of the RAD21 mRNA. 6/8 of these depleted Rad21 efficiently (at least by 90%) and resulted in similar phenotypes. After release from S-phase synchrony into medium containing nocodazole, similar to controls, the cells lacking Rad21 accumulated in mitosis. Strikingly, in almost all of these cells, all of the chromosomes had well-defined, conjoined centromeres with apparently normal primary constrictions. Even cells with hyper-condensed chromosomes remained in mitosis with conjoined centromeres, indicating that the centromere cohesion was robust. We conclude that the spindle checkpoint prevents sister centromere separation independently of the cohesin complex.

Our data extends work done previously in human cells by other authors using siRNA transfection as a means to deplete cohesin Rad21 [7], [11], [42]–[44], but based on our analysis, we reach different conclusions. The observed mitotic cells with separated sisters are likely to be the result of anaphase failures rather than from cohesion defects. We therefore propose that cells depleted of Rad21 do not separate their sister chromatids before anaphase.

In previous work a Rad21 conditional knock out cell line was generated and described in chicken DT40 cells [52]. In this case it was postulated that, after Rad21 was depleted upon tetracycline-induced repression of the remaining RAD21 gene, cohesion failed to become established in S phase. Accordingly, some mitotic chromosomes were said to have displayed weakened cohesion between sisters. Later work using the same cell line showed that the Rad21 KO DT40 cells maintain some cohesion between sisters at least until early mitosis and the conclusion was reached that cohesin is dispensable for chromosome biorientation [53].

We have reached similar conclusions based on the observation of human cells depleted of Rad21. In addition, our data are strikingly similar to those obtained through observation of mitotic chromosomes in cohesin depleted *Xenoups* egg extracts [43]. In those studies, sister chromatids that lacked bipolar attachments to the spindle maintained paired kinetochores. Moreover, based on localization of chromosomal passenger proteins, the structure of inner centromere regions appeared to be normal after depletion of *Xenopus* cohesin. Since our data are comparable with those of Kenney et al., we propose that in human cells, as in *Xenopus* egg extracts, cohesin is dispensable for the establishment of cohesion and that cohesion can be maintained at the centromere by DNA catenations at least until metaphase plate formation. We do, however, find that cohesin is required for synchronous sister chromatid separation in anaphase. Therefore cohesin functions to enhance the fidelity of chromosome segregation and may act redundantly with DNA catenations to achieve sister chromatid cohesion.

Our study has not addressed the underlying defect in Rad21-depleted cells that leads to asynchronous anaphase. In future work, two possible mechanisms should be considered. Although cytologically normal metaphase plates were observed after Rad21 depletion, we did not rule out the possibility that the bioriented chromosomes had additional incorrect microtubule-kinetochores attachments. If undetected, such persistent incorrect attachment could delay separation of some sisters relative to others, resulting in an asynchronous anaphase phenotype. Another possibility is that while DNA catenations alone are sufficient for centromere cohesion, their removal might be insufficiently controlled to allow synchronous anaphase in the absence of cohesin.

## Materials and Methods

### Cell culture and siRNA

Cells were grown at 37°C with 5% CO_2_ in DMEM containing high glucose, L-glutamine, sodium pyruvate and pyridosine hydrochloride (Gibco) plus Penicillin-Streptomycin (100 U/mL–100 mg/mL, Gibco). Cell synchronies were performed by double thymidine (2 mM) arrest and release into complete medium. The siRNA and synchrony protocols used are depicted in Figure 1. Rad21 and Smc3 were depleted using several different siRNAs (see below). siRNAs were transfected using Lipofectamine2000 (Invitrogen) following the manufacturer instructions. 6 hours after siRNA transfection, medium was changed to normal medium containing half the normal concentration of Penicillin-Streptomycin. Nocodazole was used at 0.5 µM in DMSO.

#### Cytology

For cytological analysis cells were fixed with Carnoy's and chromosome spreads prepared or with paraformaldehyde for immuno-staining, as previously described [35]. CREST antibody (Cortex Biochem) was used at 1∶500. When quantifying cellular phenotypes a minimum of 1000 cells were counted per sample. Photomicrographs were acquired with a Zeiss Axioplan2 microscope, an alpha-Plan Fluar 100x/1.45 n.a. objective, and an AxioCam MRC5 camera with Axiovision software (Zeiss).

### Western blots

Whole cell extracts were obtained and Western blots performed as previously described [35] using the following antibodies: anti-hRad21 (Abcam, 1∶1500), anti-CyclinB1 (Abcam, 1∶1500), anti-phospho-H3 (Upstate, 1∶4000), anti-alpha-tubulin (Covance, 1∶1500), anti-Apc2 (Hongtao Yu, 1∶1000), anti-Securin (Hui Zou, 1∶1000) and anti-Smc3 (Bethyl Laboratories, 1∶5000).

#### Live cell imaging

HeLa cells were plated onto poly-d-lysine coated 35 mm tissue culture dishes fitted with glass cover-slips (MatTek Cultureware). siRNA transfection and thymidine synchrony was performed as described in Fig. 1 except that upon release from the second thymidine arrest or before imaging (if imaging asynchronous cells) the standard medium containing the thymidine was exchanged for DMEM without phenol red, supplemented with 10% FBS, penicillin/streptomycin and 200 mM Trolox (Calbiochem). The dishes were transferred to a microscope humidified stage incubator containing 5% CO_2_ at 37°C. Cells were filmed at 120 second intervals with three z-sections for 16–20 hours, using a Zeiss Axiovert 200 M microscope fitted with a 40x/1.3 n.a. Plan-Neofluar objective, an Axiocam HRm camera and using Openlab software.

### siRNA sequences

siRNA sequences are shown in Table 1.

**Table 1:** siRNA Sequences

siRNA	Sequence (5–3′)	Reference
Rad21-A	GCAGACUGUAAUGAAGCAU(dTdT)	This work
Rad21-B	UAAUGAGAGAAGGCAGUGC(dTdT)	This work
Rad21-C	GCCAAGAGGAAGAGGAAGC(dTdT)	This work
Rad21-D	CAGCUUAUAAUGCCAUUAC(dTdT)	This work
Rad21-E	CAGCAGAUGCUUCAUGGUC(dTdT)	This work
Rad21-L	AUACCUUCUUGCAGACUGU(dTdT)	[11]
Rad21-M	GGAUGAUAAUGUAUCAAUG(dTdT)	Mitocheck website
Rad21-R	CGGACAUCAGGACAUCUCU(dTdT)	[42]
Rad21-W	GGUGAAAAUGGCAUUACGG(dTdT)	[7]
Smc3-p	CAGCGGUUGGCUUUAUUGC(dTdT)	This work

## Supporting Information

Figure S1 Time course of synchronous Rad21-depleted HeLa cells (Rad21-E siRNA). HeLa cells transfected (or mock transfected) with Rad21-E siRNA according to Protocol-C (see Figure 1) and released from early S-phase synchrony; samples were taken for biochemical (A) and cytological (B–K) analysis. (A) Western blot showing level of Rad21 depletion and mitotic status based on cyclin B, securin and phospho-H3 levels (Tubulin, Apc2 = loading controls). (B–D Mitotic categories scored on at least 1000 cells per cytological sample. (E–K) Cytological features of cells transfected with Rad21-E siRNA: (E) Normal sister cohesion upon nuclear envelope breakdown; (F) Normal sister cohesion in early prometaphase; (G) Normal metaphase (left cell) ; (H–I) Aberrant anaphases - centromere regions separating before arms (arrows) and some chromosomes segregating to the poles before other chromosomes have separated their sisters. (J) Abnormal telophase - chromosomes have segregated (unevenly) to three cell poles (left cell) and chromosome breaks (right cell); (K) Massive chromosome breakage.
(5.44 MB TIF)

Figure S2 Time course of synchronous Rad21-depleted HeLa cells (Rad21-L siRNA). HeLa cells transfected (or mock transfected) with Rad21-L siRNA according to Protocol-C (see Figure 1) and released from early S-phase synchrony; samples were taken for biochemical (A) and cytological (B–L) analysis. (A) Western blot showing level of Rad21 depletion and mitotic status based on cyclin B levels (Tubulin, Apc2 = loading controls). (B–D) Mitotic categories scored on at least 1000 cells per cytological sample. (E–L) Cytological features of cells transfected with Rad21-L siRNA: (E) Normal sister cohesion upon nuclear envelope breakdown; (F) Normal sister cohesion in metaphase; (G–H) Aberrant anaphases - centromere regions separating before arms (arrows) and some chromosomes segregating to the poles before other chromosomes have separated their sisters; (I–J) Abnormal telophase - chromosomes have segregated (unevenly) to more than two poles; (K) Chromosome breaks; (L) Massive chromosome breakage.
(4.58 MB TIF)

Figure S3 Time course of synchronous Smc3-depleted HeLa cells. HeLa cells transfected (or mock transfected) with SMC3-specific siRNA according to Protocol-C (see Figure 1) and released from early S-phase synchrony; samples were taken for biochemical (A) and cytological (B–K) analysis. (A) Western blot showing level of Rad21 depletion and mitotic status based on cyclin B1 and phospho-H3 levels (Apc2 = loading control). (B–D Mitotic categories scored on at least 1000 cells per cytological sample. (E–L) Cytological features of cells transfected with SMC3-specific siRNA: (E) Normal sister cohesion upon nuclear envelope breakdown; (F) Normal sister cohesion in early prophase; (G) Normal cohesion in metaphase; (H) Aberrant anaphase - centromere regions separating before arms and some chromosomes segregating to the poles before other chromosomes have separated their sisters; (I) Abnormal telophase - chromosomes have segregated (unevenly) to more than two poles; (J) Apolar telophase (right cell); (K) Massive chromosome breakage.
(5.57 MB TIF)

Figure S4 Asynchronous anaphase and chromosome breaks in cohesin-depleted HeLa cells. Cells were transfected with Rad21-L (A–H), SMC3-specific siRNA (I), SMC3- plus SMC1-sepcific siRNA (J), Rad21-W (K), or Rad21-E (L). (A) Apolar telophase cell - presumably arrested in mitosis judging by the level of chromosome condensation; (B–D) Cells in which most sister have separated and presumably segregated to the cell poles (i.e. anaphases), but some sister chromatids remain paired or are in the process of separating (arrows). (E) Apolar telophase; (F–I) Massive chromosome breakage; (J) Chromosome breakage; (K–L) Asynchronous anaphase and chromosome breakage.
(6.30 MB TIF)

Figure S5 Time lapse analysis of Rad21-E treated HeLa cells. HeLa cells expressing H2B-GFP were transfected and synchronized in early S-phase using Protocol-C (see Figure 1) and filming was initiated 4 hours after release from early S-phase (see Materials and Methods and Movie S1). (A1–7) Selected frames of a selected Rad21-depleted cell (full field movies are provided in Supplemental Material). Representative frames show: (1) Prometaphase; (2) Late prometaphase (arrow indicates a non-congressed chromosome); (3) Metaphase - chromosome must have remained cohered at their centromere regions as biorientation has been achieved; (4–6) Asynchronous onset of anaphase - some chromosomes segregating to the poles while others remain at the plate; (7) Cell becomes arrested in mitosis. Time intervals in minutes (bottom right of each frame) are shown after the start of the movie.
(0.32 MB TIF)

Movie S1 Field of Rad21-depleted cells expressing H2B-GFP (also see Fig. S5). Note the cell on the right that initially forms an apparently normal metaphase plate, spends about 1 hour in metaphase, then initiates an aberrant anaphase and becomes arrested in mitosis until the end of the movie (more that 15 hours later).
(7.72 MB MPG)
