# Supplementary figures and images for: Cohesin Is Dispensable for Centromere Cohesion in Human Cells
Source: PLoS One. 2007 Mar 28;2(3):e318. doi: 10.1371/journal.pone.0000318 (PMC1820851; doi:10.1371/journal.pone.0000318)

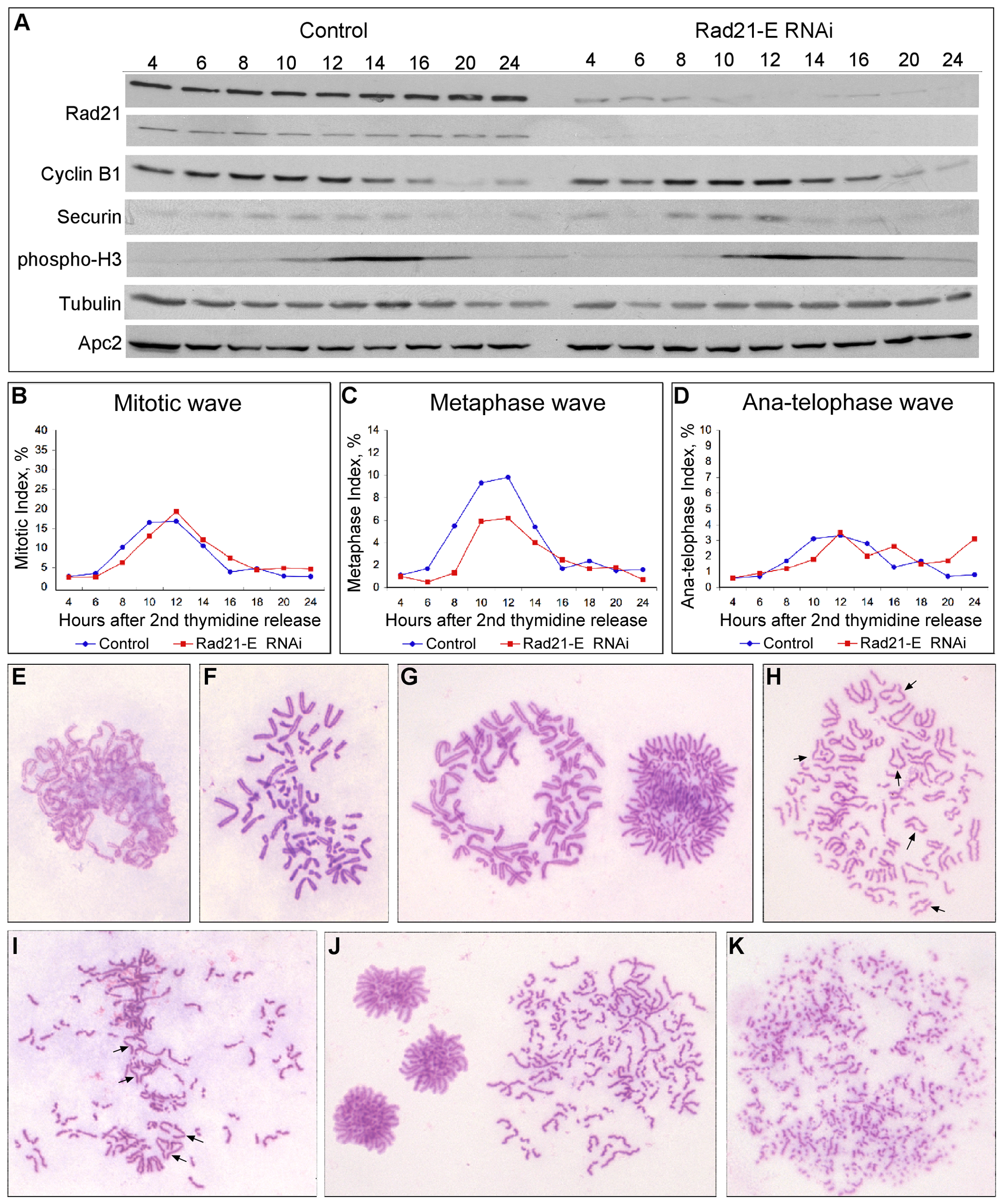

Supplement: Figure S1 — Time course of synchronous Rad21-depleted HeLa cells (Rad21-E siRNA). HeLa cells transfected (or mock transfected) with Rad21-E siRNA according to Protocol-C (see Figure 1) and released from early S-phase synchrony; samples were taken for biochemical (A) and cytological (B–K) analysis. (A) Western blot showing level of Rad21 depletion and mitotic status based on cyclin B, securin and phospho-H3 levels (Tubulin, Apc2 = loading controls). (B–D Mitotic categories scored on at least 1000 cells per cytological sample. (E–K) Cytological features of cells transfected with Rad21-E siRNA: (E) Normal sister cohesion upon nuclear envelope breakdown; (F) Normal sister cohesion in early prometaphase; (G) Normal metaphase (left cell) ; (H–I) Aberrant anaphases - centromere regions separating before arms (arrows) and some chromosomes segregating to the poles before other chromosomes have separated their sisters. (J) Abnormal telophase - chromosomes have segregated (unevenly) to three cell poles (left cell) and chromosome breaks (right cell); (K) Massive chromosome breakage. (5.44 MB TIF) [file pone.0000318.s001.tif]

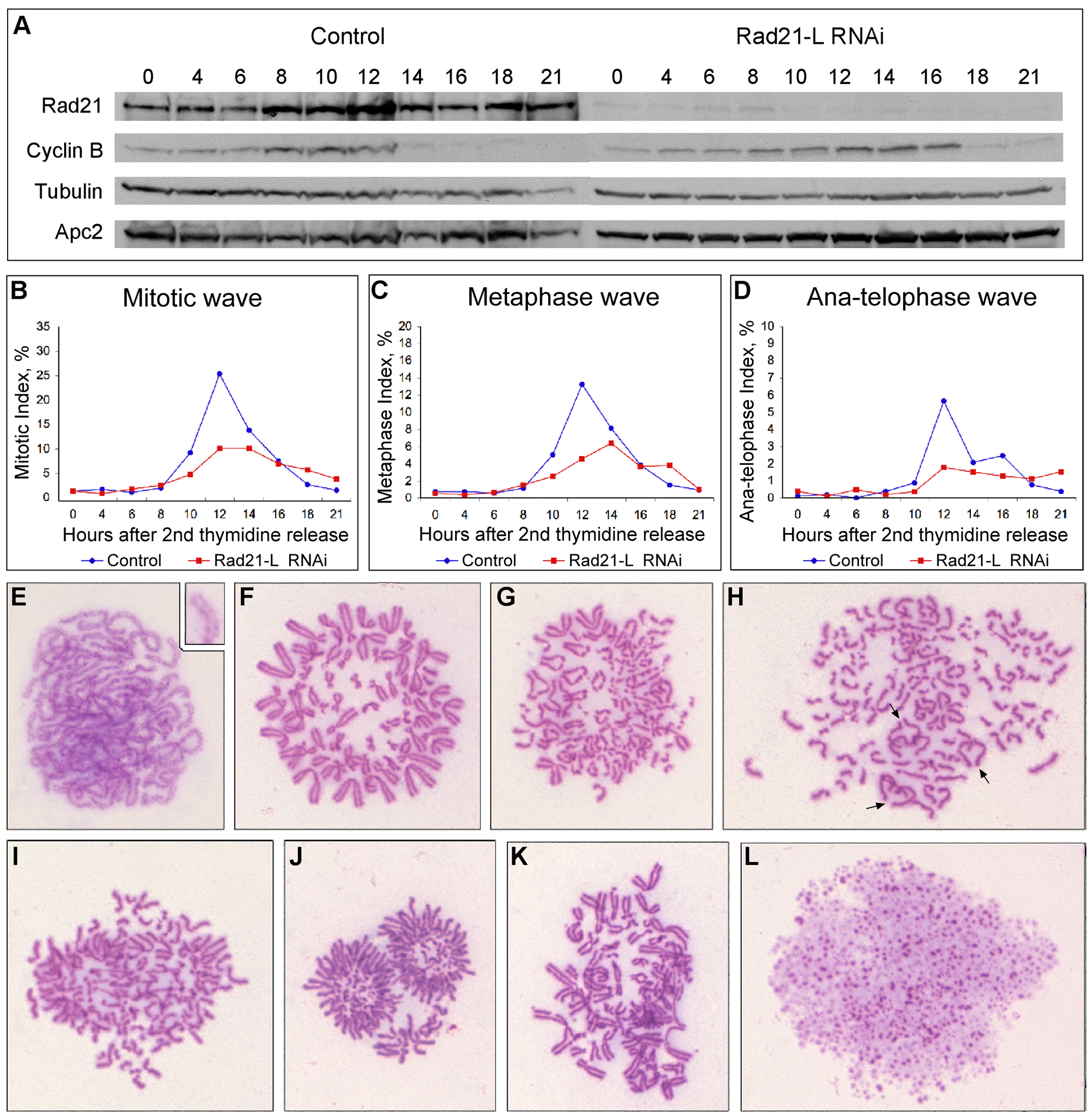

Supplement: Figure S2 — Time course of synchronous Rad21-depleted HeLa cells (Rad21-L siRNA). HeLa cells transfected (or mock transfected) with Rad21-L siRNA according to Protocol-C (see Figure 1) and released from early S-phase synchrony; samples were taken for biochemical (A) and cytological (B–L) analysis. (A) Western blot showing level of Rad21 depletion and mitotic status based on cyclin B levels (Tubulin, Apc2 = loading controls). (B–D) Mitotic categories scored on at least 1000 cells per cytological sample. (E–L) Cytological features of cells transfected with Rad21-L siRNA: (E) Normal sister cohesion upon nuclear envelope breakdown; (F) Normal sister cohesion in metaphase; (G–H) Aberrant anaphases - centromere regions separating before arms (arrows) and some chromosomes segregating to the poles before other chromosomes have separated their sisters; (I–J) Abnormal telophase - chromosomes have segregated (unevenly) to more than two poles; (K) Chromosome breaks; (L) Massive chromosome breakage. (4.58 MB TIF) [file pone.0000318.s002.tif]

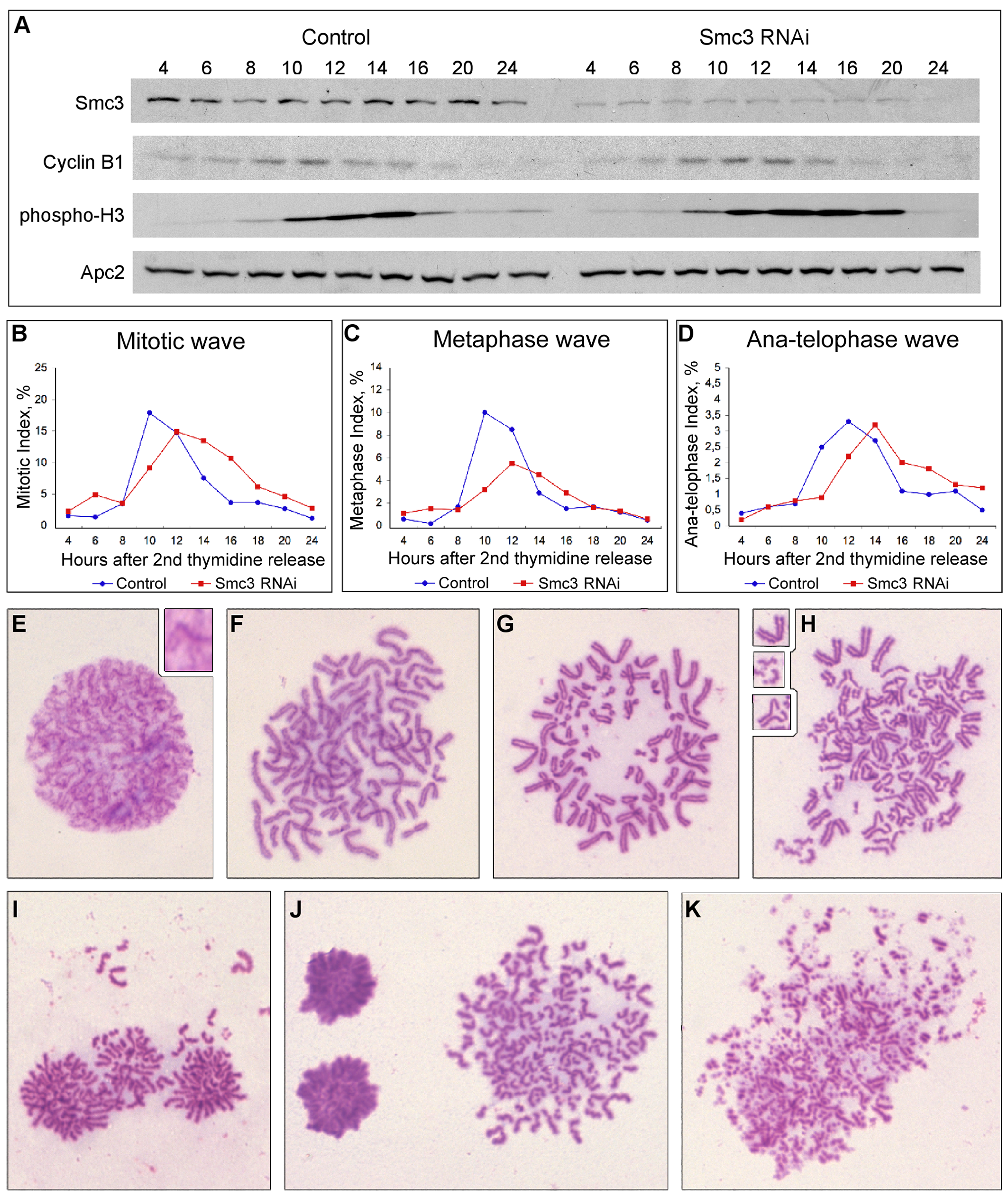

Supplement: Figure S3 — Time course of synchronous Smc3-depleted HeLa cells. HeLa cells transfected (or mock transfected) with SMC3-specific siRNA according to Protocol-C (see Figure 1) and released from early S-phase synchrony; samples were taken for biochemical (A) and cytological (B–K) analysis. (A) Western blot showing level of Rad21 depletion and mitotic status based on cyclin B1 and phospho-H3 levels (Apc2 = loading control). (B–D Mitotic categories scored on at least 1000 cells per cytological sample. (E–L) Cytological features of cells transfected with SMC3-specific siRNA: (E) Normal sister cohesion upon nuclear envelope breakdown; (F) Normal sister cohesion in early prophase; (G) Normal cohesion in metaphase; (H) Aberrant anaphase - centromere regions separating before arms and some chromosomes segregating to the poles before other chromosomes have separated their sisters; (I) Abnormal telophase - chromosomes have segregated (unevenly) to more than two poles; (J) Apolar telophase (right cell); (K) Massive chromosome breakage. (5.57 MB TIF) [file pone.0000318.s003.tif]

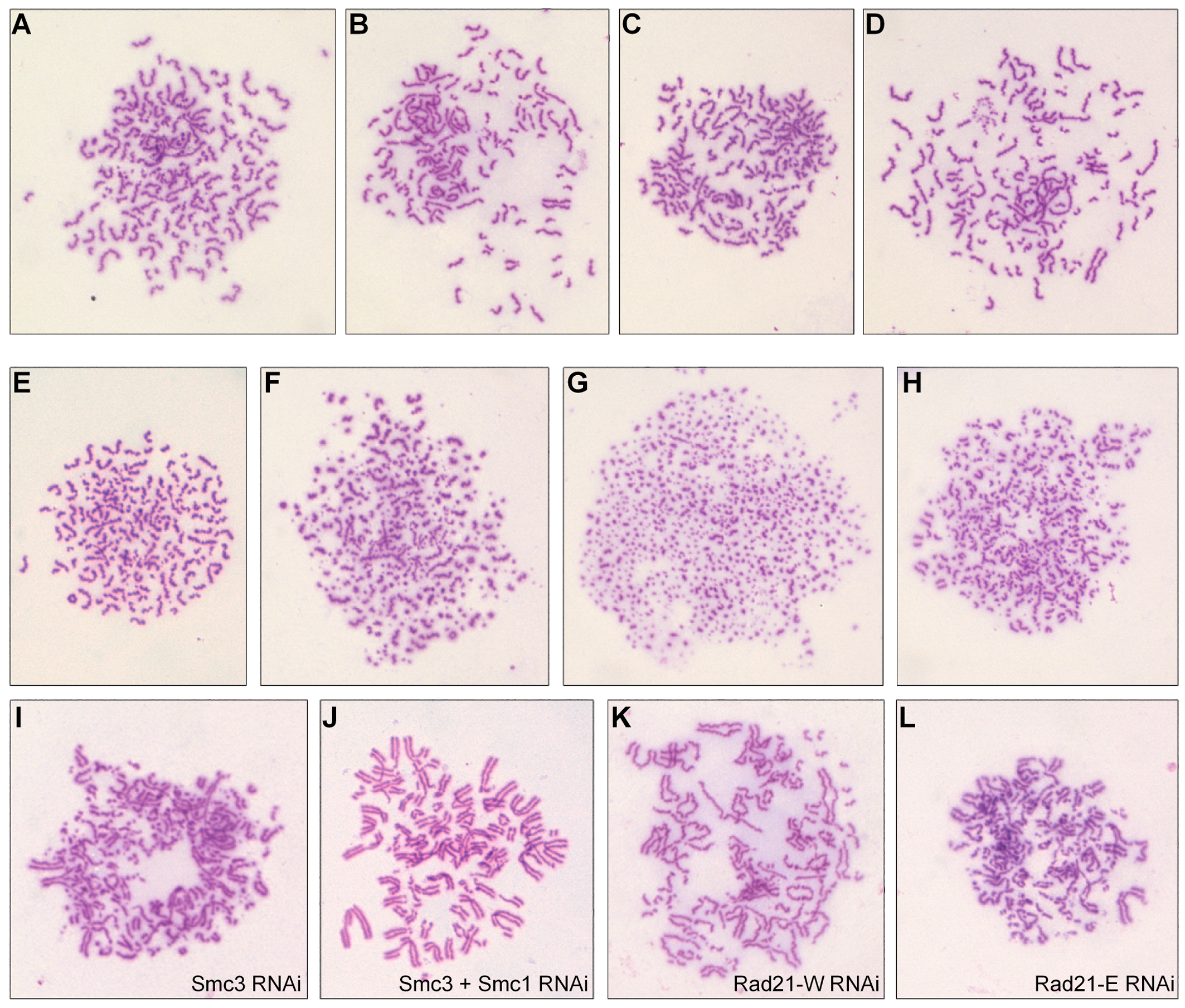

Supplement: Figure S4 — Asynchronous anaphase and chromosome breaks in cohesin-depleted HeLa cells. Cells were transfected with Rad21-L (A–H), SMC3-specific siRNA (I), SMC3- plus SMC1-sepcific siRNA (J), Rad21-W (K), or Rad21-E (L). (A) Apolar telophase cell - presumably arrested in mitosis judging by the level of chromosome condensation; (B–D) Cells in which most sister have separated and presumably segregated to the cell poles (i.e. anaphases), but some sister chromatids remain paired or are in the process of separating (arrows). (E) Apolar telophase; (F–I) Massive chromosome breakage; (J) Chromosome breakage; (K–L) Asynchronous anaphase and chromosome breakage. (6.30 MB TIF) [file pone.0000318.s004.tif]

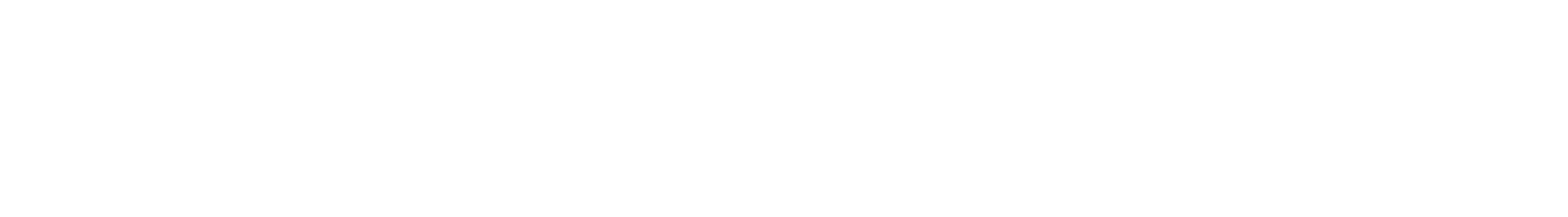

Supplement: Figure S5 — Time lapse analysis of Rad21-E treated HeLa cells. HeLa cells expressing H2B-GFP were transfected and synchronized in early S-phase using Protocol-C (see Figure 1) and filming was initiated 4 hours after release from early S-phase (see Materials and Methods and Movie S1). (A1–7) Selected frames of a selected Rad21-depleted cell (full field movies are provided in Supplemental Material). Representative frames show: (1) Prometaphase; (2) Late prometaphase (arrow indicates a non-congressed chromosome); (3) Metaphase - chromosome must have remained cohered at their centromere regions as biorientation has been achieved; (4–6) Asynchronous onset of anaphase - some chromosomes segregating to the poles while others remain at the plate; (7) Cell becomes arrested in mitosis. Time intervals in minutes (bottom right of each frame) are shown after the start of the movie. (0.32 MB TIF) [file pone.0000318.s005.tif]
